# Getting back on track to ending AIDS in children: it could just be easier than you think

**DOI:** 10.1002/jia2.26191

**Published:** 2023-11-20

**Authors:** Shaffiq Essajee, Anurita Bains

**Affiliations:** ^1^ HIV/AIDS, Health Programme Group UNICEF New York USA

1

The UNAIDS Global AIDS Strategy released in 2021 places ending inequity as the top priority to end AIDS. It recognizes the importance of a rights‐based approach to address the needs of populations that have long been neglected in the global AIDS response [1]. Among all people living with HIV, children and adolescents have faced perhaps the greatest inequities in terms of access to treatment and care.

By the end of 2022, only 57% of children living with HIV worldwide were accessing antiretroviral therapy (ART). For adolescents, treatment coverage was below 60% in every region except for Eastern and Southern Africa. By contrast among adults, treatment coverage was much higher at 77% [2]. Globally, an estimated 700,000 children (0–14 years) and 400,000 adolescents (15–19 years) living with HIV were not on ART in 2022. Across the cascade of care, from testing to treatment to viral suppression, children fared worse than adults (Table 1).

**Table 1:** The treatment cascade among adults and children living with HIV in 2022 [2]

	Adults aged 15+ years	Children aged 0–14 years
Proportion who know they are living with HIV	87%^a^	63%^a^
Proportion receiving ART	77%^a^	57%^a^
Proportion virally suppressed	72%^a^	46%^a^

Abbreviation: ART, antiretroviral therapy.

^a^ Percentages represent those who know their status/on ART/virally suppressed relative to all people living with HIV by age group.

The equity gap between adults and children has persisted since the start of the epidemic. Children with AIDS‐like illnesses were first reported in the literature almost 40 years ago, signalling that AIDS was not linked to toxins or environmental factors but caused by an infectious agent that could pass from mother to child. And yet, at almost every mile‐marker along the course of the AIDS response, children and adolescents have lagged behind adults. Lifesaving drug regimens were not approved for children for years after they were in widespread use among adults. Unlike HIV services for adults, treatment for children living with HIV continues to be centralized even in high HIV burden countries. And in most parts of the world, adults can readily access free, confidential, while‐you‐wait HIV testing, but this is not true for children and adolescents. HIV‐exposed infants need specialized diagnostic assays which are often not available or take months to process. While older children and adolescents can be tested using standard rapid tests, they depend on their caregivers to request testing and provide consent. The widening treatment gap has had dire consequences. Children aged 0–14 years contribute a full 13% of all deaths due to AIDS despite representing only 4% of all people living with HIV [2].

Focusing on children and adolescents living with HIV requires dedicated resources and tailored strategies to accelerate progress. In 2022, the Global Alliance to end AIDS in Children by 2030—a partnership with governments, networks of people living with HIV, UNICEF, WHO, UNAIDS, the Global Fund, PEPFAR and key implementers—was launched at the International AIDS Conference in Montreal. Six months later, the inaugural 12 countries* reaffirmed their shared commitment to reach the goal of ending AIDS in children in the Dar es Salaam Declaration for Action [3]. The Global Alliance is bringing much‐needed attention and visibility to the forgotten population of children and adolescents living with HIV. At the national level, governments have convened relevant stakeholders to develop Alliance Action Plans to end AIDS in children, including identifying the technical and financial resources needed to implement them. These plans mirror the four pillars of the Alliance's work: (1) identifying and treating undiagnosed children and adolescents; (2) going “the last mile” to eliminate vertical transmission of HIV to children; (3) preventing incident HIV in adolescent girls and young women who are pregnant or breastfeeding; and (4) addressing the stubborn structural barriers that hinder access to services.

All four pillars are essential elements of a sustainable and comprehensive response to end AIDS in children by 2030, and for each of them, Alliance partners have defined evidence‐based interventions for national implementation. Among these are *three strategies* that if implemented at scale, could quickly close the gaps between adults and children across the treatment cascade of testing, treatment and viral suppression and jumpstart progress towards the goal of ending AIDS in children by 2030.

**Strategy 1**

The first gap is the low number of children who know their status. Scale up of HIV testing for children and adolescents should include virologic testing of HIV‐exposed infants as well as serologic testing of older children and adolescents who may have been missed by infant Angola testing programmes. One of the most promising ways to do this is through family‐based index testing. This approach involves inviting adults living with HIV who are enrolled in care to test their children—either in facilities or in their homes or community. Several studies have shown that family‐based index testing is well accepted and results in a high “positivity” rate—even in low prevalence settings [4, 5]. In 2022, a UNICEF‐led family‐based index testing initiative in just two districts in Tanzania found 120 new cases of children living with HIV out of 866 children tested—a yield of almost 14%.† Expanded models of family‐based index testing to include children whose parent(s) may have died of AIDS‐related causes could help to ensure that additional undiagnosed HIV cases are identified, and children and adolescents are linked to treatment, care and support.

**Strategy 2**

Linking all newly diagnosed children to treatment is essential to address the second gap in the treatment cascade—the low numbers of children and adolescents living with HIV who are on ART. One important advantage of testing children in a family where other individuals are living with HIV is that parents and caregivers are more likely to ensure that children with a positive test are brought to care. In settings where paediatric HIV services remain centralized, national programmes should harness community systems to ensure that families receive the support they need to initiate children on timely treatment following a positive diagnosis.

**Strategy 3**

To address the final gap of poor rates of viral suppression among children, outdated paediatric treatment regimens must urgently be replaced with optimized therapy for all children. The approval of generic child‐friendly dolutegravir (DTG) in both single‐drug tablets and more recently fixed‐dose combinations is a significant breakthrough that could transform paediatric outcomes. To date, 89 countries are procuring paediatric DTG, and it is estimated that 62% of children on ART are receiving DTG‐containing regimens. This estimate is based on data from 17 countries which together represent two‐thirds of the global population of children on ART [6, 7]. Coverage of paediatric DTG has almost doubled since 2021 but is still far short of adult DTG coverage which is in excess of 90%. DTG‐containing regimens are clinically superior, present a higher genetic barrier to resistance and have fewer side effects than other treatment options. Rapidly transitioning all children on ART to these regimens would significantly improve paediatric treatment outcomes and rates of viral suppression.

In conclusion, while we have made considerable progress in preventing HIV in children, the gaps between adults and children living with HIV continue to widen across the treatment cascade. The reasons for this are complex but the solutions do not have to be. A concerted effort is needed now so that every child living with HIV has access to diagnosis and high‐quality treatment and care. Global cooperation, and targeted action by donors, implementers and national partners are key to ending the inequities and ultimately ending AIDS for future generations.

## COMPETING INTERESTS

The authors declare that they have no competing interests.

## AUTHORS’ CONTRIBUTIONS

The authors have contributed equally to the development of this Viewpoint.

## FUNDING

No donor or other funds were used in the writing and research for this Viewpoint.

## DISCLAIMER

The views expressed in this Viewpoint are the opinions of the authors.

## ENDNOTES

* Angola, Cameroun, Cote‐d'Ivoire, Democratic Republic of Congo, Kenya, Mozambique, Nigeria, South Africa, Tanzania, Uganda, Zambia and Zimbabwe.

† Unpublished data provided courtesy of UNICEF Tanzania.
